# Familial arthropathies

**DOI:** 10.1186/1546-0096-12-S1-I31

**Published:** 2014-09-17

**Authors:** Sulaiman Al-Mayouf

**Affiliations:** 1 Pediatric Rheumatology, King Faisal Specialist Hospital and Research Center, Alfaisal University, Riyadh, Saudi Arabia

## 

Familial arthropathy is a descriptive term, comprises a heterogeneous group of disorders. It can be either an inflammatory or a non-inflammatory condition, syndromic or non-syndromic disorder.

Juvenile idiopathic arthritis (JIA) is the most common chronic rheumatic disease in children. However, the underlying genetic background remains poorly understood. Though familial aggregation of JIA is rare, it suggests that JIA is influenced by shared genetic factors. Inheritance patterns and phenotypes probably help to clarify if familial JIA is a distinctive subtype of JIA.

Using our JIA cohort, we have identified siblings with JIA characterized by autosomal recessive transmission. Patients with familial JIA probably are different with respect to clinical and laboratory variables from sporadic JIA patients. We used linkage, homozygosity mapping and whole exome sequencing to identify the disease associated gene and underlying mutation.

It is important to remember that some patients with skeletal dysplasia and certain syndromes may present with musculoskeletal manifestations mimicking inflammatory arthritis and because of their mild phenotypes may be misdiagnosed as JIA. Careful evaluation of a child presenting with an arthropathy, particularly in a population where consanguinity is common, is required for timely and accurate diagnosis.

This presentation will give an overview of the clinical and genetic aspects of autosomal recessive JIA patients and discuss the main inherited musculoskeletal disorders including camptodactyly-arthropathy-coxa-vara-pericarditis (CACP) syndrome seen in our practice.

## Disclosure of interest

None declared.

